# Augmented reality hologram combined with pre-bent distractor enhanced the accuracy of distraction vector transfer in maxillary distraction osteogenesis, a study based on 3D printed phantoms

**DOI:** 10.3389/fsurg.2022.1018030

**Published:** 2022-11-16

**Authors:** Zongyi Yuan, Shixi He, Tianhua Jiang, Qingtiao Xie, Nuo Zhou, Xuanping Huang

**Affiliations:** ^1^ Department of Oral and Maxillofacial Surgery, College of Stomatology, Hospital of Stomatology, Guangxi Medical University, Nanning, China; ^2^ Guangxi Key Laboratory of Oral and Maxillofacial Rehabilitation and Reconstruction, Guangxi Key Laboratory of Oral and Maxillofacial Surgery Disease Treatment, Nanning, China; ^3^ Guangxi Clinical Research Center for Craniofacial Deformity, Nanning, China

**Keywords:** augmented reality, 3D printing, maxillary hypoplasia, distraction osteogenesis, distractor pre-bending, vector transfer

## Abstract

**Background:**

Vector control is a significant concern in maxillary distraction osteogenesis (DO). Distraction vector planning on the patient's 3D-printed skull phantom is more intuitive for surgeons and cost-efficient than virtual surgical planning. However, the accuracy of transferring the planned vector to intraoperative (vector transfer) according to the shape of the pre-bent footplate alone is relatively limited. The application of augmented reality (AR) in surgical navigation has been studied for years. However, few studies have focused on its role in maxillary DO vector transfer. This study aimed to evaluate the accuracy of AR surgical navigation combined with the pre-bent distractor in vector transfer by comparing it with the pre-bent distractor alone.

**Methods:**

Ten patients with maxillary hypoplasia were enrolled with consent, and three identical 3D-printed skull phantoms were manufactured based on per patient's corresponding pre-operative CT data. Among these, one phantom was for pre-operative planning (*n* = 10), while and the other two were for the AR+Pre-bending group (*n* = 10) and the Pre-bending group (*n* = 10) for the experimental surgery, respectively. In the Pre-bending group, the distraction vector was solely determined by matching the shape of footplates and maxillary surface. In the AR+Pre-bending group, the distractors were first confirmed to have no deformation. Then AR surgical navigation was applied to check and adjust the vector in addition to the steps as in the Pre-bending Group.

**Results:**

For the angular deviation of the distraction vector, the AR+Pre-bending group was significantly smaller than the Pre-bending group in spatial (*p* < 0.001), x-y plane (*p* = 0.002), and y-z plane (*p* < 0.001), and there were no significant differences in the x-z plane (*p* = 0.221). The AR+Pre-bending group was more accurate in deviations of the Euclidean distance (*p* = 0.004) and the y-axis (*p* = 0.011). In addition, the AR+Pre-bending group was more accurate for the distraction result.

**Conclusions:**

In this study based on 3D printed skull phantoms, the AR surgical navigation combined with the pre-bent distractor enhanced the accuracy of vector transfer in maxillary DO, compared with the pre-bending technique alone.

## Introduction

Distraction osteogenesis (DO) promotes new bone formation and stretches the peri-soft tissue simultaneously by slow distraction forces (1). Since the technique was introduced to treat hemifacial microsomia and maxillary hypoplasia by McCarthy et al. in 1992 and Cohen et al. in 1995, respectively, DO has played an important role in treating severe maxillary hypoplasia, especially in cases with cleft lip and palate (2–4).

The internal distractor was applied to the maxillary DO because of less physical and psychological stress (5). However, the vector cannot be modified once the distractor is activated, which can lead to unwanted distraction results (6). Therefore, reasonable pre-operative planning and accurate intraoperative transferring of distraction vectors are essential to achieve the desired outcomes. Pre-bending the commercial distractors and simulating the distraction on the patient-specific 3D-printed skull phantom was more intuitive and greatly reduced operating time. However, it seems that the accuracy of transferring the planned vector according to the shape of the pre-bent footplate alone during the intraoperative procedure was relatively limited (7, 8). Augmented reality (AR) surgical navigation allows the surgeon to see the real surgical area and the virtual preoperative plan overlapped on it simultaneously. Meanwhile, the AR hologram provides an overall view of preoperative planning information. Such technique has been used in Neurosurgery, Orthopedics, Plastic Surgery, and Oral and Maxillofacial Surgery (9–13). However, few studies focused on the effect of AR surgical navigation on vector transfer in maxillary DO.

This study aimed to assess the accuracy of AR surgical navigation combined with the pre-bent distractor for the distraction vector transfer, by comparing it with the distractor pre-bending technique alone. Our study hypothesized that AR surgical navigation combined with the pre-bent distractor would improve the vector transfer accuracy.

## Materials & methods

### Study design

**Figure 1:**
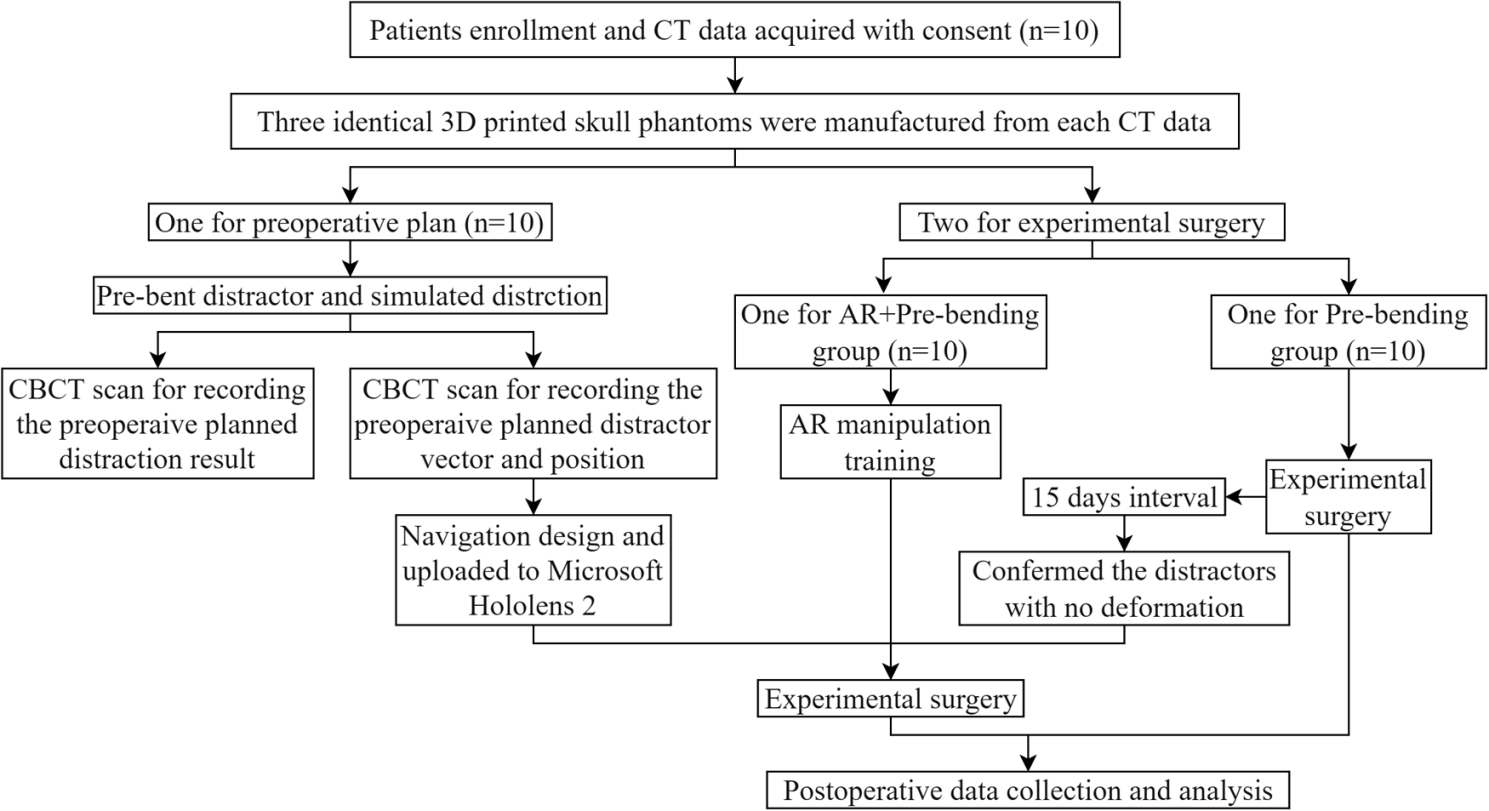
Study protocol.

This study followed the relevant provisions of the Declaration of Helsinki on human research, approved by the medical ethics committee of the Affiliated Stomatological Hospital of Guangxi Medical University, China, and registered in the China Clinical Trial Registration Center (ChiCTR2200062941). Experimental Le Fort I maxillary DO surgery was performed on 3D printed skull phantoms based on CT scans from real patients with maxillary hypoplasia. Inclusion criteria were: 1) Patients with maxillary hypoplasia and 2) Patients older than eighteen. Exclusion criteria were: 1) Patients who refused to participate in this study and 2) Patients with bilateral maxillary bone block separation due to cleft palate accompanied by alveolar cleft. Ten patients with maxillary hypoplasia were enrolled in the study. Their pre-operative CT data were used with consent to manufacture the 3D-printed skull phantoms. Three identical skull phantoms were made from each data. One phantom was for pre-operative planning (Planning Phantom, *n* = 10), while the other two were for experimental surgery (Surgery Phantom) and were assigned separately to the AR+Pre-bending group (*n* = 10) and Pre-bending group (*n* = 10). The pre-operative plans of both groups were achieved by pre-bending the distractors and simulating distraction on the planning phantoms. The vector transfer in experimental surgeries was determined solely by matching the shape of the pre-bent footplates with the maxillary surface in the Pre-bending group. In the AR+Pre-bending group, the AR surgical navigation served as a supplemental measure to check and finely adjust the position and the vector of the pre-bent distractors after the same procedure in the Pre-bending group (Figure 1).

### Phantoms

**Figure 2:**
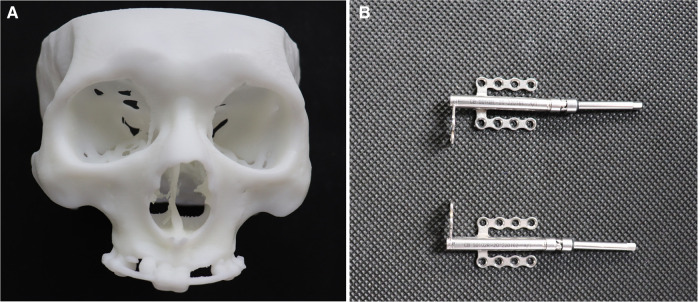
Phantom and distractors **note:** the 3D-printed skull phantom (**A**) and distractors (**B**) were applied in this study.

The pre-operative skull spiral CT scans enrolled (Siemens 256 slice dual source spiral CT, Siemens, Germany) were exported in DICOM format, and 3D reconstruction was performed using Mimics 21.0 (Materialise NV, Belgium). The 3D models were exported in stereolithography (STL) format into the Magics 24.0 (Materialise NV, Belgium) for refinement and printed with photosensitive resin using a 3D printer (Liantai lite 600, Liantai corp. Guangdong, China) with a printing layer thickness of 0.01 mm (Figure 2A).

### Pre-operative plan

**Figure 3:**
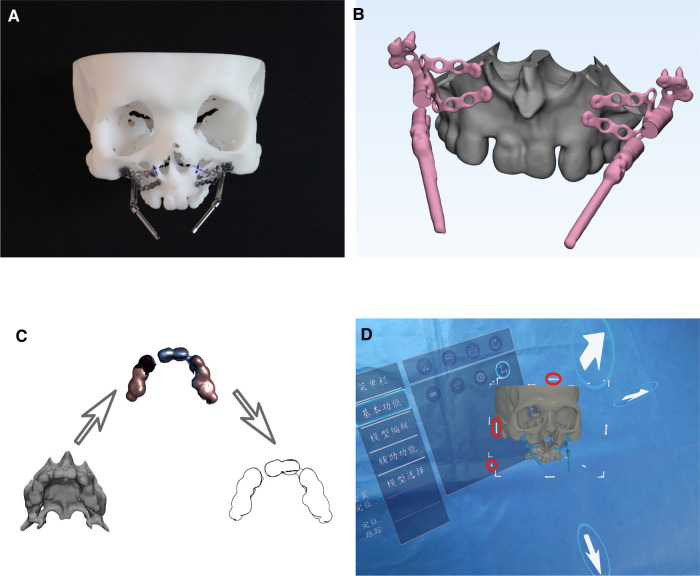
Pre-operative plan and navigation preparation **note:** (**A**) distractors pre-bending on the planning phantom. The blue marks around the osteotomy line were used to check that the distal bone block was in its original position after the osteotomy, indicating that the pre-bent distractors were not exerting unexpected pressure on the bone block. (**B**) Navigation model design. Two “windows” (black arrows) were set in the middle of bilateral driver screws as an additional visual clue to facilitate vector checking and fine adjusting. (**C**) Dentition contour extraction for automatic calibration function. (**D**) This study's “coordinate fine-tuning” function of AR surgical navigation. The three arrows allowed continuously variable translation adjustment of the hologram, while the parts marked by red ovals allowed continuously variable rotation adjustment.

On the Planning Phantom, the internal maxillary distractors (Ningbo Cibei Medical Treatment Appliance Co. Ltd, China, Figure 2B) were pre-bent and fixed with titanium screws (Ningbo Cibei Medical Treatment Appliance Co. Ltd, China) to obtain the distraction vector (Figure 3A). After Le Fort I osteotomy, a test distraction was performed according to the defined distraction distance (Supplementary material S1), to check for deviations in the maxillary midline or occlusal plane and make corresponding adjustments on vectors. Once the distraction vector was determined, an essential check and minor adjustment of the footplates were made to prevent unexpected deviation of the distal bone block from the initial position due to the potential stress of the pre-bent footplates. The Cone Beam Computer Tomography scan (CBCT, NewTom VGI, NewTom, Italy) was performed twice, one to record the planned distraction result to facilitate the postoperative analysis and the other to record the planned vector of the distractors and the position of the footplates to facilitate AR navigation designing. All the CBCT scans were saved in the DICOM format.

### Navigation preparation

The 3D models for AR navigation were reconstructed from CBCT DICOM data in the free and open-source software Slicer (version 4.11, 2020) and imported into 3-Matic 13.0 (Materialise NV, Belgium) for further navigation design. Two “windows” were set in the middle of the driver screws of the bilateral distractors to enhance depth perception by trimming the 3D models as an additional visual clue (Figure 3B). The 3D models were converted to OBJ format and imported to Unity 2021 LTS (Unity Technologies, San Francisco, United States) for compatibility with holographic navigation. On the other hand, the dentition contour of the 3D model was extracted for automatic calibration and tracking using Vuforia Engine 10.7 (Vuforia PTC Inc., Zurich, Switzerland, Figure 3C). Thus, the AR hologram of the pre-operative plan could automatically calibrate and follow the movement of the Surgery Phantom with the SLAM feature of the Microsoft HoloLens 2 (Microsoft Corp, Redmond, WA, United States). The surgical navigation program had a “coordinate fine-tuning” function which allowed fine-tuning of the hologram's position in rotation and translation after automatic calibration in case the hologram had a slightly inaccurate overlap to the surgical site (Figure 3D).

### Experimental surgery

**Figure 4:**
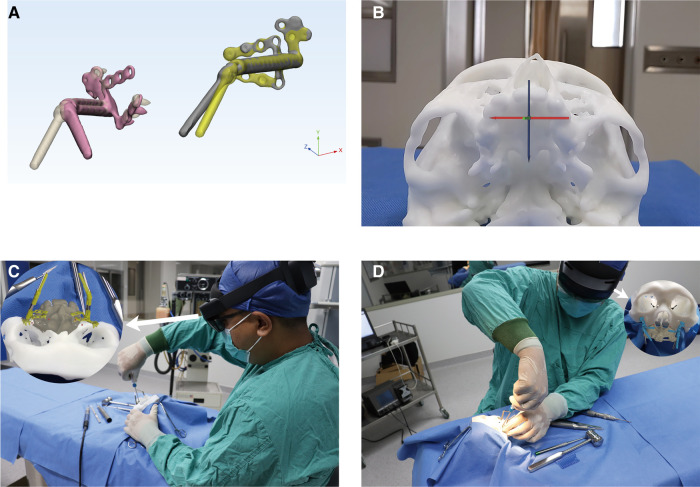
Experimental surgery **note:** (**A**) distractor shape checking after completing the Pre-bending group task, the 3D models of the actual distractors (opaque) overlapped with the pre-operative plan (translucent), indicating no deformation of the distractor occurred in the Pre-bending group task. (**B**) Automatic calibration can be achieved by the surgeon moving his head and aiming the crucifix at the dentition (Microsoft Hololens 2 view). (**C**) The surgeon checked the detractor vector in the patient's cephalic position. (**D**) The surgeon could accomplish the check in the patient's lateral position.

In a simulated operating room, all experimental maxillary DO tasks were performed by an orthognathic surgeon from the Stomatological Hospital affiliated with Guangxi Medical University. The experimental surgeries of the Pre-bending group were done first. After finishing a 15-day interval to eliminate the memory of the distractor position, the surgeon received two hours of training and practice in manipulating the AR surgical navigation and then finished the AR+Pre-bending group tasks. Three measures were implemented to maintain the consistency of the distractors in both groups: 1. The surgeon was prohibited from deforming the footplates during the experimental surgery, which was crucial. 2. Before the AR+Pre-bending group's tasks began, the AR hologram of the distractor was used to check for deformation, and the hologram of the distractor exactly overlapped with the actual one. 3. Intraoperative CBCT scans for recording distractor position and vector in the AR+Pre-bending group were also applied to compare with the pre-operative plan to check distractor for deformation. If the reconstructed distractor 3D model overlapped with the pre-operative plan, it indicated that no distractor deformation occurred after the experimental surgery was completed in the Pre-bending group (Figure 4A).

In the AR+Pre-bending group, the surgeon manipulated the Microsoft Hololens 2 by “air tapping” to open the navigation program and activate the auto-calibration function. A crucifix appeared on the screen, and the surgeon moved his head to aim the crucifix at the Surgery Phantom's dentition (Figure 4B, Supplementary materials S2, S4). Therefore, the hologram was superimposed automatically on the Surgery Phantom, and the surgeon confirmed the calibration by the overlap between the hologram and the operative area dentition, and, in case of a slight deviation, the “coordinate fine-tuning” function helped in adjusting to complete overlap. Next, after the surgeon placed the distractor by matching the pre-bent footplate shape with the maxillary surface, the distractors were checked and fine-adjusted according to the hologram (Supplementary materials S3, S4). The surgeon was allowed to perform the above operations in the cephalic or bilateral position (Figures 4C, D). The procedures remained the same as the typical maxillary DO, except once the distractors were finally fixed, the surgeon again used Microsoft HoloLens 2 to confirm the position and vector of the distractor and performed the distraction procedure according to the pre-operative plan. In the Pre-bending group, the surgeon placed the distractor according to the pre-bent footplate shape and maxillary surface without any other strategy for checking or guiding. The rest of the procedures were the same as in the AR+Pre-bending group.

### Outcome evaluations

**Figure 5:**
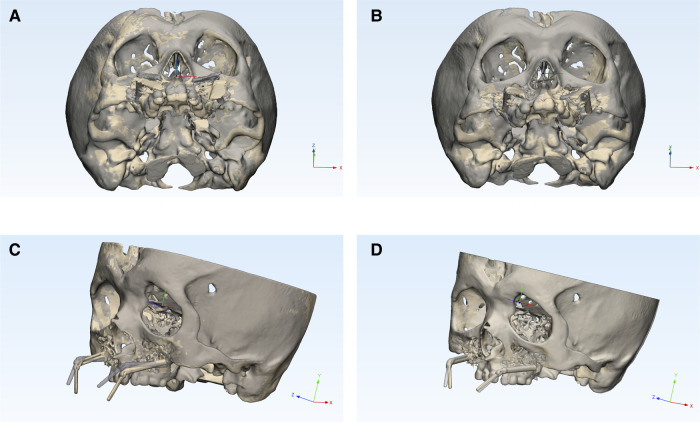
Outcome evaluation **note:** (**A**) Pre-bending group's distractor vector and position evaluation. (**B**) AR+Pre-bending group's distractor vector and position evaluation. (**C**) Pre-bending group's distraction result evaluation. (**D**) AR+Pre-bending group's distraction result evaluation. The post-operative skull model (yellow and opaque) was registered to the planned skull to assess the accuracy of the distractor vector and position (**A, B**), as well as the distraction result (**C, D**).

Post-operative CBCT scans were performed after bilateral distractors were fixed and after distraction was completed, respectively. Therefore, the intraoperative distraction vector, distractor position, and distraction result were recorded. The post-operative models reconstructed in Slicer were imported in 3-Matic (Materialise NV, Belgium). The planned skull models (accomplished in the navigation design stage) were aligned to the post-operative one using the global registration method. The post-operative models were static and served as targets (Figure 5A-D). After the registration, AR navigation accuracy was assessed by comparing the experimental surgery outcomes with the pre-operative plan. The primary outcome of this study was distraction vector accuracy. The line and the middle point between the two endpoints of the driver screw were used to define the distraction vector and the distractor position. The angular differences of the distraction vector were measured in spatial and the x-y, y-z, and z-x planes. The linear differences in the distractor position were measured in the Euclidean and three-dimensional distance, while the distraction result accuracy was evaluated by comparing the linear deviations of four landmarks (Table 1).

**Table 1:** Reference landmarks description.

Landmark	Description
ANS	Anterior nasal spine
U1	The most mesial point of the tip of the crown in between the upper central incisors.
U6l	The most inferior point of the mesial buccal cusp of the crown of the left upper molar.
U6R	The most inferior point of the mesial buccal cusp of the crown of the right upper molar.

### Statistics

Statistical analyses were performed using SPSS 26.0 (IBM Co., Armonk, NY, United States). The normality of samples was tested using the Shapiro-Wilk test. The independent t-test was applied for data that conformed to the normal distribution. Two-sample Mann-Whitney U test was applied for data not conforming to a normal distribution. A *p*-value < 0.05 was considered statistically significant.

## Results

Ten patients who met the criteria in this study with their pre-operative CT scans were enrolled, and each was applied to manufacture three same 3D-printed skull phantoms, one for pre-operative planning and two for experimental surgeries of both groups. Thirty 3D-printed skull phantoms were manufactured. The surgeon had no difficulties using AR surgical navigation, and all pre-operative plans and experimental surgeries were completed successfully. None distractors used in the Pre-bending group were deformed. The differences between the pre-operative plan and the postoperative outcomes in angular deviation of the distraction vector and linear deviation of the distractor position, along with the distraction result, are shown in Table 2. The x-y, y-z, and x-z planes represented the distractor's coronal, horizontal, and sagittal planes. The angular deviation in these planes indicated that the unplanned rotation of the distractor occurred in the corresponding planes. The x, y, and z-axis represented the horizontal, vertical, and anteroposterior directions, respectively. The linear deviation in these axes indicated that the unplanned translation of the distractor or the maxillary bone block occurred along the corresponding axis.

**Table 2:** Differences between the two groups in angular deviations of the vector and linear deviations of the distractor position.

Variable	AR+Pre-bending Group	Pre-bending Group	*P* value
Angular deviation of the distraction vector (°)			
Spatial***	2.19 (1.03, 3.03)	5.03 (3.58, 7.88)	<0.001
x-y plane**	3.36 (1.21, 5.51)	7.24 (2.93, 14.25)	0.002
y-z plane***	1.03 (0.52, 2.67)	3.66 (2.09, 7.29)	<0.001
x-z plane	1.10 (0.49, 1.94)	1.95 (0.37, 4.96)	0.221
Liner deviation of the distractor position (mm)			
In Euclidean distance**	0.89 (0.67, 1.45)	1.70 (0.87, 2.97)	0.004
x-axis	0.46 (0.14, 0.78)	0.69 (0.31, 3.45)	0.052
y-axis*	0.48 (0.19, 0.83)	0.82 (0.56, 1.78)	0.011
z-axis	0.34 (0.13, 0.68)	0.61 (0.26, 0.98)	0.096

**Note:** Values were presented as median (P25, P75).

* indicates *p* < 0.05.

** indicates *p* < 0.01.

*** indicates *p* < 0.001.

For the angular deviation of the distraction vector, the AR+Pre-bending group result was smaller than the Pre-bending group in spatial (2.19 [1.03, 3.03°] vs. 5.03 [3.58, 7.88°], *p* < 0.001), x-y plane (3.36 [1.21, 5.51°] vs. 7.24 [2.93, 14.25°], *p* = 0.002) and y-z plane (1.03 [0.52, 2.67°] vs. 3.66 [2.09, 7.29°] *p* < 0.001), while no statistical difference was found in the x-z plane (Table 2).

The linear deviation of the distractor position in the AR+Pre-bending group was smaller than the Pre-bending group in Euclidean distance (0.89 [0.67, 1.45 mm] vs. 1.70 [0.87, 2.97 mm], *p* = 0.004) and in the y-axis (0.48 [0.19, 0.83 mm] vs. 0.82 [0.56, 1.78 mm], *p* = 0.011). No statistical differences were found in the×or y-axis (Table 2).

**Figure 6:**
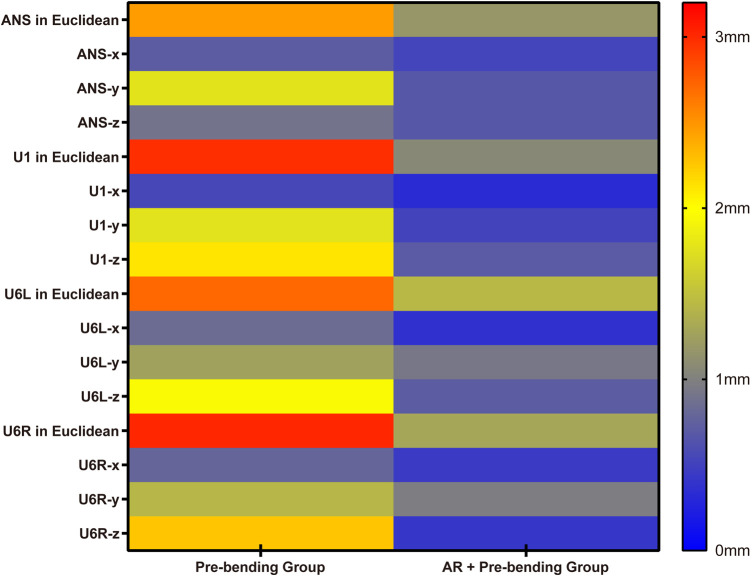
Differences between the two groups in linear deviations of the distraction result **note:** ANS in Euclidean, ANS-x, ANS-y, and ANS-z, respectively, represented the linear deviation in Euclidean distance, x, y, and z-axes of landmark ANS. U1 in Euclidean, U1-x, U1-y, and U1-z, respectively, represented the linear deviation in Euclidean distance, x, y, and z-axes of landmark U1. U6l in Euclidean, U6l-x, U6l-y, and U6l-z, respectively, represented the linear deviation in Euclidean distance, x, y, and z-axes of landmark U6l. U6R in Euclidean, U6R-x, U6R-y, and U6R-z, respectively, represented the linear deviation in Euclidean distance, x, y, and z-axes of landmark U6R. The vertical gradient color strip located on the right side of the figure shows the color represented in distance error levels, from 0 mm (blue) to 3.2 mm (red), and the baseline is 2 mm (yellow), which represents clinically acceptable error.

For the distraction results, the linear deviation of the AR+Pre-bending group was smaller than the Pre-bending group in Euclidean distance in all landmarks, ANS (1.17±0.68 vs. 2.46±1.41 mm, *p* = 0.018; 95% CI: −1.29 [-2.33, −0.25 mm]); U1 (1.06±0.55 vs. 2.98±1.36 mm, *p* = 0.001; 95% CI: −1.91 [−2.93, −0.90 mm]); U6l (1.44 ± 0.28 vs. 2.71 ± 1.17 mm, *p* = 0.007; 95% CI: −1.27 [-2.12, −0.43 mm]); U6R (1.30±0.39 vs. 3.02±1.13 mm, *p* = 0.001; 95% CI: −1.72 [-2.55, −0.89 mm]). The linear deviation of the AR+Pre-bending group was smaller than the Pre-bending group in the y and z-axes of the landmark U1, the x and z-axes of the landmark U6l, and the x, y, and z-axes of the landmark U6R (Figure 6).

## Discussion

This study aimed to evaluate the AR surgical navigation accuracy as a supplemental checking and adjusting measure combined with the pre-bent distractor on transferring the pre-operatively planned distraction vector to intraoperative by conducting the experimental maxillary DO surgeries on the 3D printed skull phantoms. The AR surgical navigation combined with the pre-bent distractor showed a smaller angular deviation of the distraction vector and minor linear deviations of the distractor position and distraction result.

**Figure 7:**
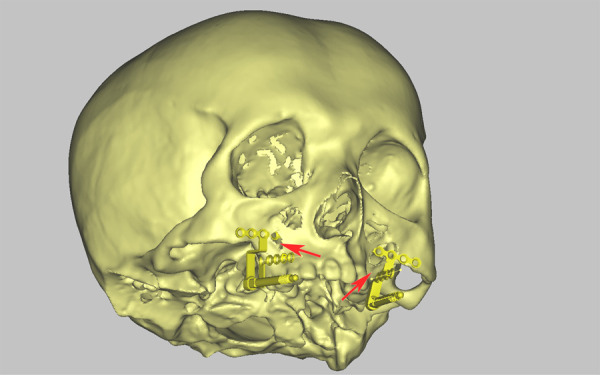
Abnormal position of distractors in VSP **note:** the limitation of VSP for distraction osteogenesis in maxillary hypoplasia, the virtual footplate could not be contoured to match the profile of the maxilla, which led to the abnormal position of the footplate (red arrow).

The internal distractor vector control is a major concern in DO (14). The effect of virtual surgical planning (VSP) combined with CAD/CAM splints has been demonstrated in several studies (15–17). However, the virtual footplates could not be contoured to conform to the 3D models, and the requirement of strong 3D visualization capabilities increased the difficulty of establishing the required vectors of movement during the VSP procedure (Figure 7) (18, 19). On the contrary, pre-bending footplates and simulating distraction on the 3D-printed patient skull phantom were more intuitive for surgeons (19–21). The CAD/CAM splint's low cost-efficient and excessive tissue dissection for its placement were another two issues that limited its clinical application (22–24). Therefore, the presented study focused on the role of AR surgical navigation in transferring the preoperatively planned vector to the intraoperative.

In a study of mandibular DO on goats, conventional surgical navigation (the monitor was outside the surgical area) played a crucial role in obtaining satisfying distraction results by transferring the planned drilling hole position of the footplate intraoperatively. Although the accuracy of the vector control was not evaluated, it demonstrated the possibility of a virtual reference for navigating the DO procedure (25).

Another study showed evidence for the feasibility of the AR hologram as DO surgical navigation. The surgeon completed the mandibular osteotomy along the virtual osteotomy plane shown on the hologram. The post-operative CT scan confirmed that the actual osteotomy plane was in line with the pre-operative plan (11); however, few studies focused on the role of AR surgical navigation in vector transferring of maxillary DO. In contrast to the mandible, the maxilla has a more complex surface morphology and, in most cases, requires bilateral distraction, indicating that the vector control in the maxilla is more complex.

Our study indicated that the proposed AR surgical navigation combined with pre-bent distractors enhanced the accuracy of vector and position transferring in maxillary DO, compared with that by pre-bent distractors alone. As the vectors were transferred more accurately to intraoperative, the linear deviations of the phantom maxilla distraction result in the AR+Pre-bending group were smaller than those in the Pre-bending group. Badiali et al. emphasized the importance of vector transfer in the distraction result, where conventional surgical navigation was applied to transfer the vector. They evaluated the angular and linear deviation of the vector and distractor, respectively, through the data recorded by intraoperative navigation devices (26).

In this study, the corresponding deviations were smaller because the operative area and navigation hologram were integrated into the same field of view by the Microsoft HoloLens 2. Therefore, the surgeon had better hand-eye coordination. The position relationship between the driver screw and its hologram was judged properly using additional visual clues set on the bilateral distractor hologram, consistent with De Paolis et al. (27). In another study of mandibular DO on the standard mandibular 3D printed phantoms, a series of new fully customized distraction assemblies were developed, including the individualized 3D printed titanium footplate, customized driver screw, and the CAD/CAM splint for guiding the former two devices into position. The linear deviations of the distraction results demonstrated the proposed assembly's accuracy (15). On the contrary, these linear deviations in this study were smaller. It was probably because the total errors generated from the AR surgical navigation design and calibration procedure were smaller than those generated from the design and manufacturing procedure of the assemblies.

In this study, the angular deviations in the x-y and y-z planes were significantly smaller in the AR+Pre-bending group, while as shown in the distraction result, the linear deviation of landmark U1 and U6R in the y and z-axes and U6l in the z-axis were also smaller. It was because the angular deviation in the y-z plane has the greatest influence on the distraction results, since it indicates an unplanned rotation along the sagittal plane of the driver screw, and it might finally lead to the linear deviation of the maxillary block along the y-axis or the z-axis. The angular deviations of the vector in the x-z plane and linear deviations of the distraction result in the x-axis of landmark ANS, and U1 were similar between the two groups, while the linear deviations in the x-axis of landmark U6l and U6R were smaller in the AR+Pre-bending group. It was because the range of angular deviation in the x-z plane was smaller, leading to a smaller deviation of the bilateral resultant vector in the x-z plane. Therefore, a smaller linear deviation of the distraction result occurred in the AR+Pre-bending group. On the other hand, the asymmetrical distraction direction on both sides resulted in a tendency of the distal bone (maxilla) to rotate. Due to the distractor applied in this study, the center of the rotation was in the maxilla anterior part. Therefore, the difference in the linear deviation in the posterior landmark U6l and U6R between the two groups was more significant. Despite the angular deviation in the x-y plane representing the driver screw rotating along the long axis, which may not affect the direction of the distraction, and the linear deviation of the distractor position is less critical, the errors were minor in the AR+Pre-bending group, indicating that AR surgical navigation had a positive effect (26).

The method proposed in this study had some features that could contribute to clinical application. Automatic calibration was completed within 1 min, and dentation was selected as the calibration target since the contours of the hard tissue were more favorable to automatic calibration. Meanwhile, the “fine-tuning of coordinates” function in the proposed AR surgical navigation ensured the calibration accuracy, although it would take a few extra minutes, and with experience, it would be shorter. In preoperative planning, determining the vector and predicting distraction results did not require sophisticated CAD design experience for surgeons, and it was without the restriction of the distractor type. During the intraoperative, the proposed AR surgical navigation can also be used to check distractors intraoperatively to identify the possible deformation during sterilization in time. In addition, the “air tapping” and “voice control” functions enabled sterile manipulation of the Microsoft HoloLens 2 (28). The 3D hologram superimposed on the surgical area offered the surgeon an excellent spatial awareness of the pre-operative plan (29). Lastly, the 3D-printed skull phantom for the preoperative plan has been demonstrated to be cost-efficient and was cheap as US$30 in this study, and a Microsoft HoloLens 2 only cost US$3,500, which was beneficial in less developed areas (30).

There were a few limitations to the study. First, a larger number of cases in further study may be considered to obtain more convincible statistical results due to the limited cases. Second, considering the influencing factors in real surgery, such as soft tissue compression, real patients-based maxillary DO procedures are needed to further validate the clinical feasibility of this technology. Third, the learning curve for the design of the hologram and the use of AR for surgical navigation should be evaluated in future work. At last, the navigation design in this study required different software with a limited number of functions. In the future, software needs to be developed to integrate the required functionality, which will also help further reduce the cost.

## Conclusions

In the experimental surgeries based on the 3D printed skull phantoms of the patients, the AR surgical navigation combined with the pre-bent distractor enhanced the accuracy of vector transfer in maxillary DO, compared with the pre-bending technique alone. Thus, our study suggests that AR surgical navigation combined with the pre-bent distractor may be a low-cost and efficient protocol for vector transferring of the maxillary DO.

## Data Availability

The raw data supporting the conclusions of this article will be made available by the authors, without undue reservation.
