# Supplementary material for: Augmented reality hologram combined with pre-bent distractor enhanced the accuracy of distraction vector transfer in maxillary distraction osteogenesis, a study based on 3D printed phantoms
Source: Front Surg. 2022 Nov 16;9:1018030. doi: 10.3389/fsurg.2022.1018030 (PMC9709275; doi:10.3389/fsurg.2022.1018030)
Supplement: Supplementary file 1 [file Table1.docx]

| Case | Defined distraction distance（mm） | |
| --- | --- | --- |
|  | Left driver screw | Right driver screw |
| 1 | 4 | 4 |
| 2 | 6 | 6 |
| 3 | 6 | 6 |
| 4 | 6.4 | 6.4 |
| 5 | 8 | 8 |
| 6 | 6 | 8 |
| 7 | 6 | 10 |
| 8 | 7.2 | 7.2 |
| 9 | 12 | 12 |
| 10 | 6 | 6 |

Supplementary material 1: The defined distraction distance
