# Supplementary material for: Augmented reality hologram combined with pre-bent distractor enhanced the accuracy of distraction vector transfer in maxillary distraction osteogenesis, a study based on 3D printed phantoms
Source: Front Surg. 2022 Nov 16;9:1018030. doi: 10.3389/fsurg.2022.1018030 (PMC9709275; doi:10.3389/fsurg.2022.1018030)
Supplement: Supplementary file 4 [file Datasheet1.docx]

**Video Manual**

**Video 1.** Demonstration of the calibration & tracking process and the “coordinate fine-tuning” function.

**Note:** After the corresponding hologram was selected, the auto-calibration & tracking function was activated. Then, the surgeon aligned the crucifix (displayed on the screen) to the dentition, and the hologram automatically overlapped with the skull phantom. The “coordinate fine-tuning” was applied to the translation (horizontal, vertical, and anterior-posterior) and rotation (Yaw, Pitch, and Roll) adjustment if slight non-overlap of the hologram was detected.

**Video 2.** Demonstration of the Augmented Reality hologram-assisted vector control.

**Note:** The position and vector of the distractor were determined by matching the pre-bent footplate with the maxillary surface first. Then the distraction vector and the distractor position were further adjusted according to the hologram.

Please noted that the videos were recorded by the built-in camera and recording function of the HoloLens, which showed the hologram better. However, due to the camera hardware, as well as the fact that the hologram calibration and tracking occupied the HoloLens camera resource simultaneously, there were some discrepancies between the recorded videos and the surgeon’s actual observation:

1. At the beginning of calibration, the surgeon aimed the crucifix at the dentition instead of the upper-left area of the phantom shown in supplementary material 1, video 1.

2. The hologram was superimposed steadily to the phantom after calibration, instead of the hologram slowly moving from the upper-left to the phantom as shown in supplementary material 1, video 1.

3. The robustness of the hologram was better when video recording was not active at the same time.
